# Biological Safety of Fish (Tilapia) Collagen

**DOI:** 10.1155/2014/630757

**Published:** 2014-04-07

**Authors:** Kohei Yamamoto, Kazunari Igawa, Kouji Sugimoto, Yuu Yoshizawa, Kajiro Yanagiguchi, Takeshi Ikeda, Shizuka Yamada, Yoshihiko Hayashi

**Affiliations:** Department of Cariology, Nagasaki University Graduate School of Biomedical Sciences, Nagasaki 852-8588, Japan

## Abstract

Marine collagen derived from fish scales, skin, and bone has been widely investigated for application as a scaffold and carrier due to its bioactive properties, including excellent biocompatibility, low antigenicity, and high biodegradability and cell growth potential. Fish type I collagen is an effective material as a biodegradable scaffold or spacer replicating the natural extracellular matrix, which serves to spatially organize cells, providing them with environmental signals and directing site-specific cellular regulation. This study was conducted to confirm the safety of fish (tilapia) atelocollagen for use in clinical application. We performed *in vitro* and *in vivo* biological studies of medical materials to investigate the safety of fish collagen. The extract of fish collagen gel was examined to clarify its sterility. All present sterility tests concerning bacteria and viruses (including endotoxin) yielded negative results, and all evaluations of cell toxicity, sensitization, chromosomal aberrations, intracutaneous reactions, acute systemic toxicity, pyrogenic reactions, and hemolysis were negative according to the criteria of the ISO and the Ministry of Health, Labour and Welfare of Japan. The present study demonstrated that atelocollagen prepared from tilapia is a promising biomaterial for use as a scaffold in regenerative medicine.

## 1. Introduction

Regenerative medicine consists of three components: cells, nutrients (growth factors, cytokines, and chemicals, etc.), and scaffold materials [1]. The combined application of these components is important. With respect to scaffold manufacturing, the use of bioactive natural organic materials originating from marine products is indispensable, as severe infections (zoonosis), including bovine spongiform encephalopathy, avian and swine influenza, and tooth-and-mouth disease in bovines, pigs, and buffalo, occur worldwide. Marine collagen derived from fish scales, skin, and bone has been widely investigated for its potential application as a scaffold material and carrier due to its bioactive properties, such as excellent biocompatibility, low antigenicity, and high biodegradability and cell growth potential [2, 3]. As fish collagen (FC) generally has a low degenerative temperature (*T*_d_), it melts when placed in contact with the human body for clinical application, which renders this biomaterial difficult to handle* in vivo* at actual physical temperatures used in human medical applications. The low stability of FC is thought to be due to its low hydroxyproline content compared to that observed in bovine collagen [4]. Recently, our laboratory showed that the *T*_d_ of collagen extracted and purified from the skin of a tropical fish, tilapia, is 35-36°C (unpublished data). Therefore, tilapia collagen is believed to be a powerful candidate for use in making a clinical scaffold as an alternative to bovine collagen.

The aim of this study was to confirm the safety of FC for use in clinical application. We performed* in vitro* and* in vivo* biological examinations of medical materials to investigate the safety of FC. FC is used as a solution in order to promote stem cells suspension. Therefore, the extract of FC gel was examined to clarify its sterility. In addition, biological studies of low concentrations of FC solution were conducted in accordance with ISO standards.

## 2. Materials and Methods

### 2.1. Preparation of FC

Fish type I atelocollagen produced by solubilized tilapia skin was kindly supplied by Nippi Inc., Biomatrix Institute (Ibaragi, Japan). A total of 0.1% FC dissolved in 1.5-fold concentrated PBS (−) (pH 7.4) was used for the following biological experiments.

### 2.2. Sterility Test

#### 2.2.1. Aerobic and Anaerobic Bacteria Denial Tests

Two mL of 0.1% FC was added to a 90 mm culture dish. FC solution was gelled at 37°C for 30 minutes in an atmosphere of 5% CO_2_ and air. Ten mL/dish of PBS (−) was added on FC gel and cultured at 37°C for three days in a CO_2_ incubator (SCA-165DS, ASTEC Co., Ltd., Fukuoka, Japan). After three days of culture, PBS (−) was decanted into special culture vials containing medium for aerobic (BD BACTEC Plus Aerobic/F, BD and Company, Sparks, MD, USA) or anaerobic (BD BACTEC Plus+ Anaerobic/F, BD and Company) bacteria, respectively. The vials were cultured for five days and analyzed using a totally automatic blood culture testing apparatus (BACTEC 9240/9120, BD and Company, Franklin Lakes, NJ, USA).

#### 2.2.2. Fungi Denial Test

A total of 0.4 mL of 0.1% FC was added to a 35 mm culture dish. FC solution was gelled at 37°C for 30 minutes in an atmosphere of 5% CO_2_ and air. Two mL/dish of PBS (−) was added on FC gel and cultured at 37°C for three days in a CO_2_ incubator. After five days of culture, PBS (−) was decanted into a special glass tube containing medium for fungi (Sabouraud 2 agar (SAB2-T), BioMerieux, Marcy l'Etoile, France). The tube was cultured for five days and analyzed using a totally automatic blood culture testing apparatus (BACTEC 9240/9120, BD and Company, Franklin Lakes, NJ, USA).

#### 2.2.3. Endotoxin Denial Test

A total of 0.4 mL of 0.1% FC was added to a 35 mm culture dish. FC solution was gelled at 37°C for 30 minutes in an atmosphere of 5% CO_2_ and air. Two mL/dish of PBS (−) was added on FC gel and cultured at 37°C for three days in a CO_2_ incubator. After three days of culture, PBS (−) was mixed with limulus reagent (Wako Pure Chemical Industries Ltd., Osaka, Japan) and analyzed according to a turbidimetric time assay using a Wako Toxinometer (MT-5500, Wako Pure Chemical Industries Ltd.). A negative result was defined as a level below 1.0 pg/mL.

#### 2.2.4. Mycoplasma and Virus Denial Test

Three mL of 0.1% FC was added to a 90 mm culture dish. FC solution was gelled at 37°C for 30 minutes in an atmosphere of 5% CO_2_ and air. NOS-1 cells were seeded at a density of 1 × 10^5^ cells on FC gel with 15 mL of *α*-MEM containing 10% fetal bovine serum and cultured at 37°C for five days in a CO_2_ incubator. *α*-MEM decanted from the culture dish was assayed for the presence of mycoplasma, and NOS-1 cells retrieved from the surface of the FC gel by adding 2 mL of trypsin-EDTA to the culture dish and pipetting in *α*-MEM were analyzed for the presence of viruses using real-time PCR (Applied Biosystems 7900HT, Life Technologies Corp., Carlsbad, CA, USA).

### 2.3. Biological Evaluations of Fish Collagen

#### 2.3.1. Cell Toxicity Test (ISO 10993-5:2009)

In order to evaluate the cell toxicity of FC, V79 cells (a cell line consisting of fibroblasts derived from the lungs of male Chinese hamsters) were used to inhibit colony-formation according to the direct contact method. One mL of the cell suspension (40 cells/mL) was seeded in a 24-well culture plate and cultured for seven days. The following six groups were designed for comparison: (1) control (culture medium: MEM 10) group, (2) negative control material (plastic sheet) group, (3) positive control material (polyurethane containing 0.25% zinc (ZDBC)) group, (4) experimental (fish collagen gel (for gelation: 37°C, 30 minutes)) group, (5) control (DMSO: solvent for ZDBC) group, and (6) positive control (ZDBC) group. After seven days of culture, the culture medium was removed. The cells were fixed with 1 mL of 100% methanol for five minutes and were stained with 4% Giemsa stain solution (Merck KGaA, Darmstadt, Germany). The mean number of colonies (*n* = 4) was counted by the naked eye and was converted to a percentage of the total number of colonies (100%) in the control group. Cell toxicity was defined as a colony-formation ratio below 30% in the FC group.

#### 2.3.2. Sensitization Test (ISO 10993-10:2010) (Figure 1)

Sensitization to FC was investigated using a guinea pig maximization test (GPMT) [5]. Ten Scl:Hartley male guinea pigs (5 weeks of age) were used. A total of 0.1 mL/site of 0.1% FC with Freund's complete adjuvant (FCA) was injected intracutaneously into the back of the scapula in the experimental group. Seven days after intracutaneous sensitization, 10 w/w% sodium lauryl sulfate ointment as an inflammatory chemical was openly applied to the same sensitized site, then removed after 24 hours. Subsequently, 0.2 mL/site of 0.1% FC was sensitized for 48 hours according to the close application method. Fourteen days after sensitization, 0.1 mL/site of either 0.1% FC or control sterile water was closely applied for 24 hours in the bilateral flank to induce inflammation. The applied site was observed 24 and 48 hours after the application of FC or water. In the negative control group, five guinea pigs were used, in which the injection water was injected intracutaneously and applied for sensitization in order to induce inflammation. In the positive control group, five guinea pigs were used, in which 0.1 w/w% 2,4-dinitrochlorobenzene was injected intracutaneously and applied for sensitization in order to induce inflammation. At 24 and 48 hours after the induction, the skin reaction (erythema and swelling) was observed by the naked eye and judged according to the criteria proposed by Magnusson and Kligman [5].

The Magnusson and Kligman criteria [5]:

0: no reaction,
1: discrete or porphyritic erythema,
2: moderately fused erythema,
3: extremely severe erythema and swelling.

#### 2.3.3. Chromosomal Aberration Test (ISO 10993-3:2003)

In order to evaluate the induction of chromosomal aberrations with FC application, CHL/IU cells (a cell line consisting of fibroblasts derived from the lungs of new-born female Chinese hamsters) were treated with a short-term method under the presence or absence of metabolic activation and a 24-hour continuous method under the absence of metabolic activation. PBS (−) was used as the negative control material, and mitomycin C (under the absence of metabolic activation) and cyclophosphamide monohydrate (under the presence of metabolic activation) were used as the positive controls. The volume in the experimental group was determined according to the “OECD 473,* in vitro* mammalian chromosome aberration test.” The chromosomal structure and number of cells exhibiting aberration were examined at three observation doses of FC: 1.3, 2.5, and 5 *μ*L/mL. The CHL/IU cells were seeded in 60 mm culture dishes at a density of 5 × 10^4^ cells and were cultured in 10% bovine calf serum-MEM for 72 hours. The number of cells was counted using a hemocytometer under a phase-contrast microscope. Population doubling (PD) of cells was defined as

(1) $$\begin{array}{c} \text{PD}=\frac{\left[\log\left(N/X_{0}\right)\right]}{\log2}, \end{array}$$

where  *X*_0_  is vital cell number (mean) at the start of the treatment and  *N*  is vital cell number (mean) after treatment (6 hours later).

The cell proliferation ratio (%) was converted to a percentage of the PD (100%) in the negative control. The inhibition of PD was defined as a cell proliferation ratio below 50%.

A 0.05 mL aliquot of 10 *μ*g/mL colcemid (Life Technologies Corp., CA, USA) was added into the culture medium (final concentration: 0.1 *μ*g/mL) two hours before the end of the culture period. The 5 mL of 0.075 mol/L KCL was added to the cell suspension for 30 minutes for hypotonic treatment at 37°C. After the cells were fixed with Carnoy's fixative (methanol : acetic acid = 3 : 1) for 30 minutes at 4°C, two drops of cell floating solution were dropped on the slide glass. The specimen was dried at room temperature and was stained with 3.0% Giemsa stain solution (pH 6.8, Merck KGaA) for 15 minutes.

*Chromosomal Aberration Was Classified Based on the Following Criteria [6]*

 Structural aberrations:
   chromatid breaks,
   chromatid exchange,
   chromosome breaks,
   chromosome exchange,
   others.
 Numerical aberrations:
   polyploidy,
   endoreduplication.

The differences between the treatment and negative control groups were compared using Fisher's exact test (Bonferroni correction).

#### 2.3.4. Intracutaneous Test (ISO 10993-10:2010)

FC gel (for gelation: 37°C, 30 minutes) was extracted in physiological saline or sesame oil for 72 hours at 37°C. The extract was used to investigate intracutaneous irritability. A total of 0.2 mL/site of each extract was injected intracutaneously at five sites in the back in five Japanese white male rabbits. The sites of injection were observed at 24, 48, and 72 hours after treatment. In the control group, extract solvents (physiological saline and sesame oil) were injected using similar methods to those applied in the experimental group. The criteria for scoring were as follows.

- * Erythema and eschar:*
- no erythema,
- very slight erythema,
- clear erythema,
- moderate or severe erythema,
- severe erythema (beet redness) to mild crust formation (deep wound).
- * Edema;*
- no edema,
- very slight edema,
- slight edema (clear swelling),
- moderate edema (approximately 1 mm in diameter),
- severe edema (over 1 mm in diameter).

The score was defined as follows.

Total scores (A + B) at 24, 48, and 72 hours after treatment were divided by 15 (three (observation periods) by five (injected sites)).

The final score was obtained as follows.

The calculated score was divided by three (the number of animals). When the difference value (the mean score of the experimental group minus that of the control group) was below 1.0, the examined sample was judged to be negative.

#### 2.3.5. Acute Systemic Toxicity Test (ISO 10993-11:2006)

The extraction method applied to the FC gel was the same as that used in the intracutaneous test. The extract was used to investigate acute systemic toxicity. Each extract (50 mL/kg) was injected once into the caudal vein (physiological saline) or abdominal cavity (sesame oil) in five Crlj:CD1 male mice. In the control group (five mice), extract solvents (physiological saline and sesame oil) were injected using similar methods to those applied in the experimental group. The animals were observed at 4, 24, 48, and 72 hours after injection.

*The Criteria Used to Assess Acute Systemic Toxicity.*

 Negative findings.

There were no differences in the biological response in the experimental group compared to the control animals during all observation periods.

 Positive findings.
  1. More than two animals died after exposure to the experimental extract.
  2. More than two animals showed reactions associated with toxicity, such as convulsions and weakening, after exposure to the experimental extract.
  3. More than three animals showed a more than 10% weight loss after treatment with the experimental extract.

#### 2.3.6. Pyrogenic Test (ISO 10993-11:2006)

The extraction method applied to the FC gel was the same as that used in the intradermal test. Extract dissolved in physiological saline was used to investigate the presence of endotoxic and nonendotoxic pyrogens. The extract (10 mL/kg) was injected once into the auricular vein in three Japanese white male rabbits. The rectal temperature was measured six times at 30 minute intervals for three hours after injection. The increase in temperature was determined based on the difference between the highest temperature after injection and the control temperature. The sample was judged to be negative when the total increase in body temperature in three animals was below 1.3°C [7]. The sample was judged to be positive when the total increase in body temperature in three animals was over 2.5°C [7].

#### 2.3.7. Hemolysis Test (ISO 10993-4:2002)

The extraction method applied to the FC gel was the same as that used in the intracutaneous test. Extract dissolved in physiological saline was used to investigate hemolytic toxicity. Blood samples (0.1 mL) not containing fibrin were prepared from two Japanese white rabbits and added to the extract (5 mL). The mixture was incubated for one, two, and four hours at 37°C. The mixture was then spun using a centrifuge (EX-126, Tomy Seiko Co., Ltd., Tokyo, Japan) at 750 ×g for five minutes at 4°C. The supernatant was collected, and the absorbance (Ab) was read at 576 nm using an ultraviolet and visible spectrophotometer (UV-1600, Shimadzu Co., Kyoto, Japan). The hemolysis index (HI) (%) was defined as

(2) HI  (%)=((Ab  of  experimental  sample− Ab  of  negative  control  sample)  ×(Ab  of  positive  control  sample− Ab  of  negative  control  sample)−1)×100.

*The Criteria Used to Define Hemolysis [7]*

 HI ≤ 2: none.
 2 < HI ≤ 10: slight.
 10 < HI ≤ 20: moderate.
 20 < HI ≤ 40: severe.
 40 < HI: very severe.

#### 2.3.8. Three-Dimensional (3D) Cell Culture (*N* = 3)

Osteoblasts (NOS-1 cells [8]) derived from human osteosarcoma were seeded in a 65 mm culture dish at a density of 1 × 10^5^ cells in *α*-MEM containing 10% fetal bovine serum and cultured in a humidified incubator at 37°C in an atmosphere of 5% CO_2_ and air. The subconfluent monolayer was passaged via three-dimensional experiments conducted in a 4-well plate (1.9 cm^2^ growth surface/well, NUNC, Thermo Fisher Scientific Inc., Roskilde, Denmark). First, the wells were precoated with a thin FC layer to prevent cell migration by adding 250 *μ*L of 0.1% FC solution and incubating the mixture at 37°C for 30 minutes to enable gelation. Second, the same volume of FC solution was added to the gel bed. Third, NOS-1 cells were trypsinized (trypsin-EDTA, Gibco Lab) and resuspended with *α*-MEM. A total of 4.0 × 10^4^ cells were mixed with FC solution and incubated at 37°C for 15 minutes to enable gelation. Finally, 500 *μ*L of mineralization medium (supplemented with 10^−7^ M dexamethasone, 10 mM *β*-glycerophosphate, and 50 *μ*g/mL of ascorbate 2-phosphate) or normal *α*-MEM was added to the FD gel. All samples were prepared in triplicate and cultured in a humidified incubator at 37°C in an atmosphere of 5% CO_2_ and air. The medium was changed every three days. After 14 days of culture, the cells were washed with PBS (−), fixed with 100% methanol, and stained with 1% alizarin red S (Sigma-Aldrich Co., St. Louis, MO, USA).

## 3. Results

### 3.1. Sterility Test

Negative results (not detected) were obtained in all sterility tests for bacteria, mycoplasma, viruses, and endotoxin (below 1.0 pg/mL) in 0.1% FC solution (*n* = 2).

### 3.2. Biological Evaluations of Fish Collagen

#### 3.2.1. Cell Toxicity Test (Table 1)

The colony-formation ratio of cells seeded directly on FC gel was 93.9% and that observed in the negative and positive control groups was 96.8% and 0.0%, respectively. FC did not inhibit colony-formation, as the FC ratio was over 30%. The colony-forming ratio in the positive control group decreased dose-dependently, and the concentration at 50% inhibition of colony-formation (IC_50_) was 1.60 *μ*g/mL.

#### 3.2.2. Sensitization Test

Neither an abnormal general condition nor changes in weight were observed in any animals during the experimental periods. Following sensitization with intracutaneous and topical application of 0.1% FC, no skin reactions were observed at the sites treated with 0.1% FC or control water to induce inflammation during the observation period in any of the 10 animals (Table 2). No skin reactions were noted at any of the sites in the five negative control animals. However, clear skin reactions (erythema and/or swelling) were detected during the observation period in all five positive control animals.

#### 3.2.3. Chromosomal Aberration Test (Figures 2 and 3)

No color changes in the medium, which occurred as a result of changes in pH, were observed after adding FC. The rate of cell proliferation calculated using population doubling did not decrease under either treatment condition (over 87%). The incidence of structural aberrations without metabolic activation after the application of 1.3, 2.5, and 5 *μ*L/mL (short-term treatment) was 1.0, 0.0 and 0.0%, respectively (the negative control: 0.0%). The incidence with metabolic activation after the application of 1.3, 2.5, and 5 *μ*L/mL (short-term treatment) was 0.0, 0.5, and 1.0%, respectively (the negative control: 0.5%). The incidence after the application of 1.3, 2.5, and 5 *μ*L/mL (continuous treatment) was 0.5, 1.0, and 0.0%, respectively (the negative control: 0.0%). There were no significant differences in the chromosomal structures of cells exhibiting aberrations in the experimental group under either treatment condition compared to that observed in the negative control group (*P* > 0.05).

The incidence of numerical aberrations without metabolic activation after the application of 1.3, 2.5, and 5 *μ*L/mL (short-term treatment) was 0.0% in all groups (the negative control: 0.0%). The incidence with metabolic activation after the application of 1.3, 2.5, and 5 *μ*L/mL (short-term treatment) was 0.0% in all groups (the negative control: 0.0%). The incidence after the application of 1.3, 2.5, and 5 *μ*L/mL (continuous treatment) was 0.0, 1.0, and 0.5%, respectively (the negative control: 0.0%). There were no significant differences in the number of cells exhibiting chromosomal aberrations in the experimental group under either treatment condition compared to that observed in the negative control group (*P* > 0.05).

#### 3.2.4. Intracutaneous Test (Table 3)

Neither death, an abnormal general condition, nor any change in weight was observed in any of the animals during the experimental period. No skin reactions (erythema or swelling) were noted after injection of the extracts in physiological saline (mean score: 0.00) or control solution (mean score: 0.00) during the observation period in the experimental or control animals. Skin reactions were observed after injection of the extracts in sesame oil as follows: score-1 erythema at all five sites in three animals and score-1 edema at one or two sites in two animals at 24, 48, and 72 hours after injection. The total score for erythema/eschar and edema was 3.60 (mean: 1.20) from 24 to 72 hours after injection. Skin reactions were observed after injection of the control sesame oil as follows: score-1 erythema at all five sites in three animals and score-1 edema at three or four sites in two animals at 24 and 48 hours after injection. Furthermore, skin reactions were also observed after injection of the control sesame oil as follows: score-1 erythema at all five sites in three animals and score-1 edema at two or four sites in two animals at 72 hours after injection. The total score for erythema/eschar and edema was 4.33 (mean: 1.44) from 24 to 72 hours after injection. The difference value (1.20 minus 1.44: −0.24) was below 1.0.

#### 3.2.5. Acute Systemic Toxicity Test

No deaths, a normal general condition or normal changes in weight, and normal antopsy findings were observed in the experimental or control animals.

#### 3.2.6. Pyrogenic Test (Table 4)

No changes in weight or a normal general condition were observed in any of the animals throughout the experimental period. The total increase in temperature was 1.15°C at 30 minutes, 1 hour, 1 hour and 30 minutes, 2 hours, 2 hours and 30 minutes, and 3 hours after injection of FC extract in physiological saline. Therefore, FC was found to be a nonpyrogenic material.

#### 3.2.7. Hemolytic Test

The mean HI in two animals injected with FC extract in physiological saline was 0.2% after one hour of incubation, 0.4% after two hours of incubation, and 1.0% after four hours of incubation. The HI was below 2% during all incubation periods. Therefore, FC was found to be a nonhemolytic material.

#### 3.2.8. 3*D* Cell Culture

**Figure 4:**
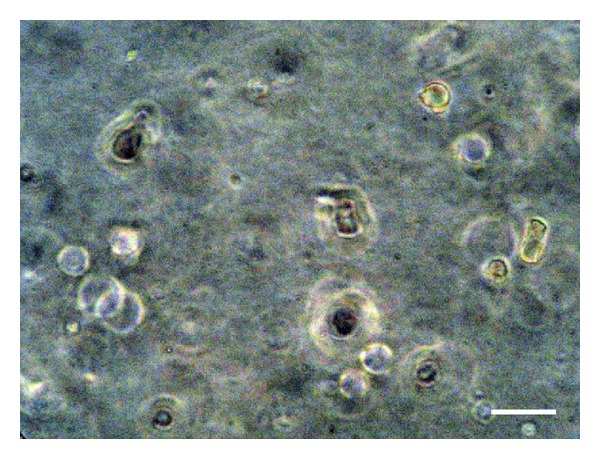
The NOS-1 cells maintained a globular shape in the FC gel cultured in the mineralization medium at seven days after seeding. Scale bar = 15 *μ*m.

**Figure 5:**
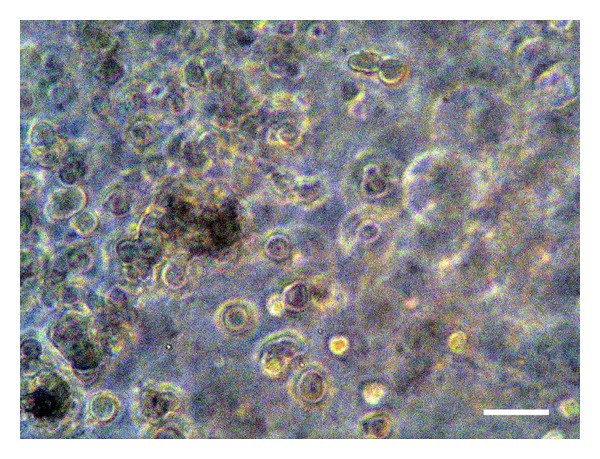
The NOS-1 cells exhibited a grape cluster, consisting of a stratified structure in the FC gel cultured in the mineralization medium at 14 days after seeding. Scale bar = 15 *μ*m.

**Figure 6:**
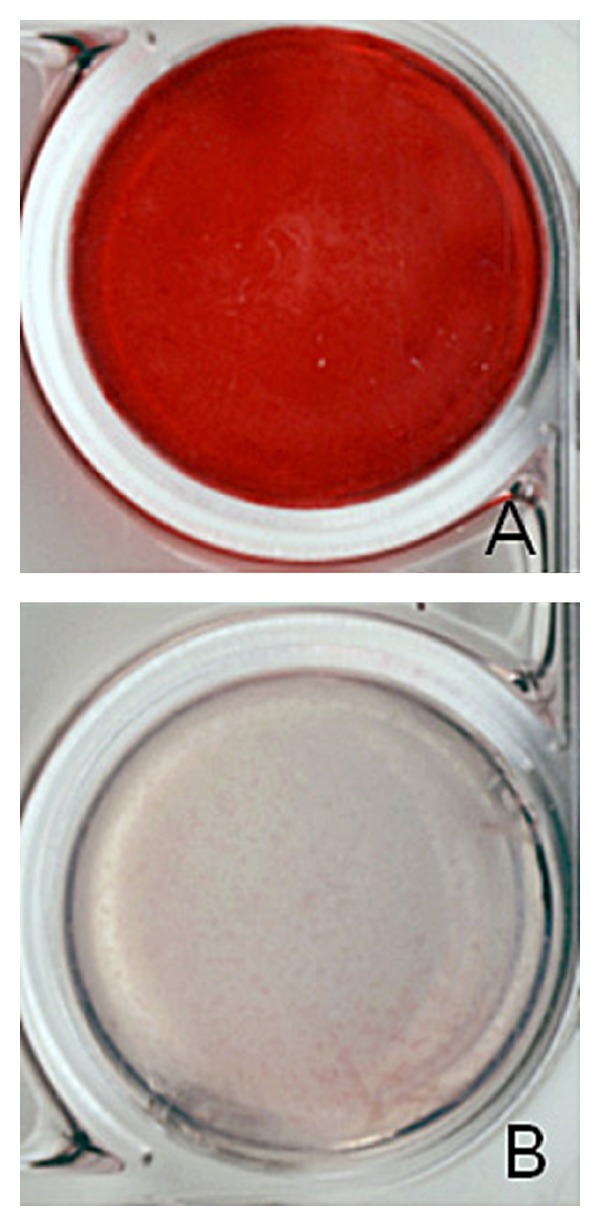
A representative photograph of the extracellular matrix mineralization in the FC gel (A). Note the lack of staining of the extracellular matrix in the control medium (B).

The NOS-1 cells grew while maintaining a globular shape in the FC gel (Figures 4 and 5). The cells proliferated inside the FC gel according to the number of seeded cells for 14 days in both media. The cells in the control medium grew rapidly compared to those cultured in the mineralization medium. Alizarin red S staining clearly demonstrated extracellular matrix mineralization among the cells cultured in the mineralization medium (Figure 6).

## 4. Discussion

The FC supplied by the manufacturer was prepared via extraction using pepsin in acetic acid and filtrated with a 0.45 *μ*m filter (personal communication). The FC used in this study can be stably supplied year round. The final concentration was prepared on a clean bench in our laboratory. All present sterility tests of bacteria and viruses (including endotoxin) were negative. Therefore, the process of FC preparation is considered acceptable for clinical application. With respect to conservation of FC, the preliminary study indicated that the FC was stable for over six months under frozen conditions at −20°C. The frozen FC was kept in a refrigerator for four to five hours before use.

The main reason for using collagen is its excellent biocompatibility, low antigenicity [9], and high direct cell adhesion properties and biodegradability compared to chitin/chitosan and synthetic polymers [10]. This is the first study to investigate the biological safety of FC in accordance with ISO standards. All tests for cell toxicity, sensitization, chromosomal aberrations, intracutaneous reactions, acute systemic toxicity, pyrogenic reactions, and hemolysis were negative according to the criteria of the ISO and the Ministry of Health, Labour and Welfare of Japan. These results are due to the long-term sterility of the material, the completion of gelation over 15 minutes at 37°C, and the proper and sufficient ateloconditions. The short-term gelation of FC solution is also suitable for extraction tests. Fish type I collagen is an effective material for a biodegradable scaffold or spacer replicating the natural extracellular matrix, which serves to spatially organize cells, providing them with environmental signals and directing site-specific cellular regulation [11, 12].

3D cultures are more physiologically relevant than conventional 2D cultures based on the seeding of cells on plastic dishes [13, 14]. As a result, 3D cultures enable the study of physiological processes that cannot be examined in 2D cultures, including differentiation [15] and morphogenesis [16, 17]. The 3D cell culture system used in this study clearly showed a high level of direct cell adhesion with FC gel as a scaffold with a greater degree of osteoblastic differentiation for mineralization, which indicates a high possibility for the clinical application of FC.

In conclusion, although further studies are required, particularly experiments using large animals, to evaluate the clinical suitability of FC, the atelocollagen prepared from tilapia is a strongly promising biomaterial for use as a scaffold in regenerative medicine.

## Figures and Tables

**Figure 1:**
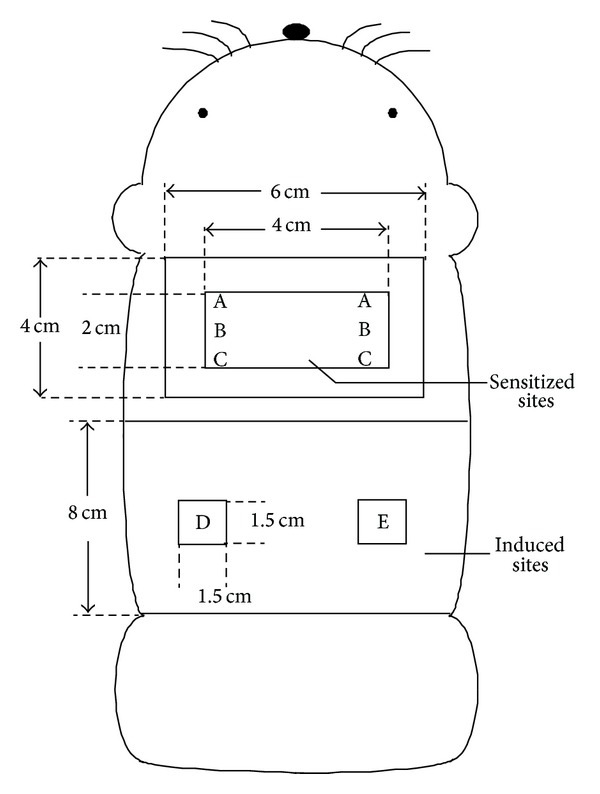
A schematic representation of sensitized (A, B, and C) and induced (D and E) sites in GPMT. A: water + FCA. B: FC. C: FC (two times concentrated) + FCA. D and E: close application sites.

**Figure 2:**
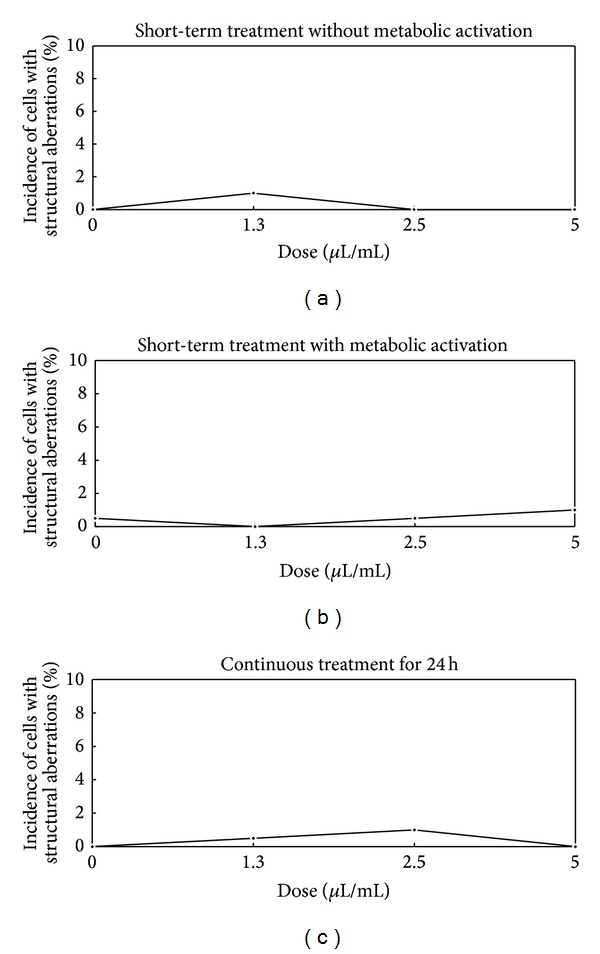
Dose-response curves (structural aberrations of chromosome) following short-term and continuous treatments.

**Figure 3:**
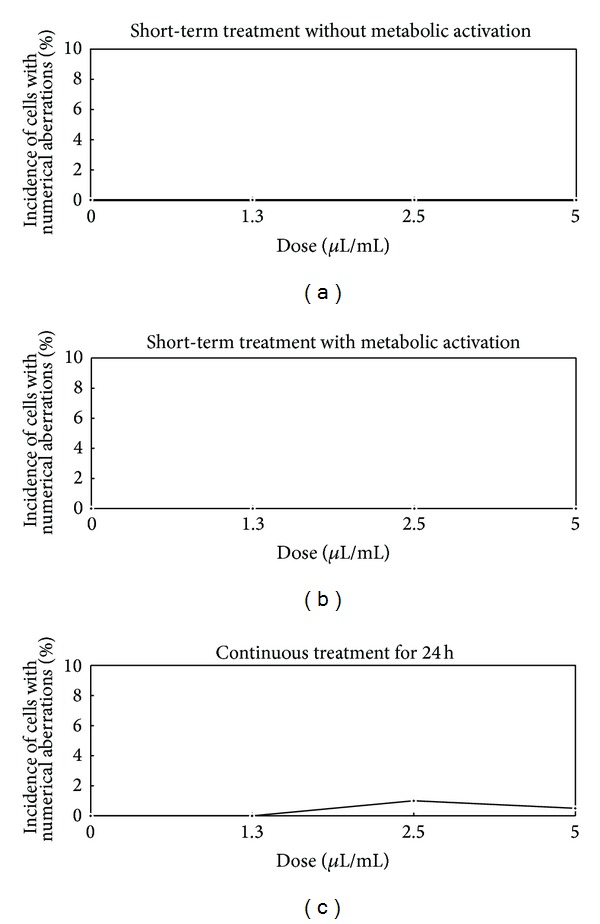
Dose-response curves (numerical aberrations of chromosome) following short-term and continuous treatments.

**Table:** (a)

Test area	Colonies/well	Mean ± SD	Colony-formation ratio (%)
Control (MEM10 medium)	38, 43, 42, 40	40.8 ± 2.2	100
Negative control material			
Plastic sheet, toluene resisting	36, 40, 41, 41	39.5 ± 2.4	96.8
Positive control material B			
Polyurethane film containing 0.25% ZDBC	0, 0, 0, 0	0.0 ± 0.0	0.0
Test article			
Fish collagen	38, 40, 36, 39	38.3 ± 1.7	93.9

**Table:** (b)

Test solution	Concentration (*μ*g/mL)	Colonies/well	Mean ± SD	Colony-formation ratio (%)	IC_50_ (*μ*g/mL)
Control (DMSO)	0	41, 37, 41, 44	40.8 ± 2.9	100	—

Positive control article ZDBC	1	37, 43, 37, 41	39.5 ± 3.0	96.8	1.60
	2	5, 11, 6, 6	7.0 ± 2.7	17.2	
	3	0, 0, 0, 0	0.0 ± 0.0	0.0	

ZDBC: Zinc dibutyldithiocarbamate.

DMSO: Dimethyl sulfoxide.

**Table 2:** Individual score of the skin reaction on challenge sites.

Group	Animal no.	Treatment			Grading scale*	
		Induction		Challenge	24 hr**	48 hr**
		Intradermal injection	Topical application			
2	6	Fish collagen (0.1%)	Fish collagen (0.1%)	Fish collagen (0.1%)	0	0
				Water for injection	0	0
	7	Fish collagen (0.1%)	Fish collagen (0.1%)	Fish collagen (0.1%)	0	0
				Water for injection	0	0
	8	Fish collagen (0.1%)	Fish collagen (0.1%)	Fish collagen (0.1%)	0	0
				Water for injection	0	0
	9	Fish collagen (0.1%)	Fish collagen (0.1%)	Fish collagen (0.1%)	0	0
				Water for injection	0	0
	10	Fish collagen (0.1%)	Fish collagen (0.1%)	Fish collagen (0.1%)	0	0
				Water for injection	0	0

Notes: *Grading scale. Patch test reaction:

0: no reaction,

1: discrete or porphyritic erythema,

2: moderately fused erythema,

3: extremely severe erythema and swelling.

**Time (hours) after challenge.

**Table 3:** Irritation score (intracutaneous) in male rabbits.

Animal no.	Test and control articles*	Total				Total score	Irritation**score ∑(*A* + *B*)/15	Total irritation score	Mean^#^	Difference of mean^##^
		Pre	Time (hour) after administration					
			24	48	72			
1 2 3	Physiological saline extract of Fish collagen	0 0 0	0 0 0	0 0 0	0 0 0	0 0 0	0.00 0.00 0.00	0.00	0.00	0.00
1 2 3	Physiological saline extract (control)	0 0 0	0 0 0	0 0 0	0 0 0	0 0 0	0.00 0.00 0.00	0.00	0.00	

1 2 3	Sesame oil extract of Fish collargen	0 0 0	6 5 7	6 5 7	6 5 7	18 15 21	1.20 1.00 1.40	3.60	1.20	−0.24
1 2 3	Sesame oil extract (control)	0 0 0	5 8 9	5 8 9	5 7 9	15 23 27	1.00 1.53 1.80	4.33	1.44	

Notes: *Each test and control article was administered to 5 sites per animal.

**Total score [erythema and eschar formation (*A*) and edema formation (*B*) of 24, 48, and 72 hours after administration of per animal]/15 [3 grading periods (24, 48, and 72 hours after administration) × 5 administered sites].

^#^Total irritation score/3 animals.

^##^Mean (each test article) − Mean (each control article).

**Table 4:** Body temperature in male rabbits.

Test article	Animal no.	Rectal temperature (°C)									Temperature rise (°C)	Total^#^ (°C)	Pyrogenicity
		Before administration		Control temperature	After administration					
		1st	2nd		3rd	4th	5th	6th	7th	8th
Physiological saline extract of fish collagen	1	38.8	38.7	38.75	38.9	39.1	39.2	39.2	39.2	39.2	0.45	1.15	−
	2	39.0	39.0	39.00	39.3	39.4	39.4	39.4	39.3	39.2	0.40		
	3	38.7	38.7	38.70	38.9	38.8	38.8	39.0	39.0	39.0	0.30		

Notes: ^#^The total of the temperature rise.

−: Negative.
